# Effects of syntaxins 2, 3, and 4 on rat and human epithelial sodium channel (ENaC) in *Xenopus laevis* oocytes

**DOI:** 10.1007/s00424-020-02365-6

**Published:** 2020-03-27

**Authors:** Robert Rauh, Fabian Frost, Christoph Korbmacher

**Affiliations:** Institut für Zelluläre und Molekulare Physiologie, Friedrich-Alexander-Universität Erlangen-Nürnberg (FAU), Waldstrasse 6, 91054 Erlangen, Germany

**Keywords:** Epithelial sodium channel (ENaC), SNARE, Trafficking, *Xenopus laevis* oocyte expression system, Electrophysiology

## Abstract

Syntaxins are SNARE proteins and may play a role in epithelial sodium channel (ENaC) trafficking. The aim of the present study was to investigate the effects of syntaxin 2 (STX2), syntaxin 3 (STX3), and syntaxin 4 (STX4) on rat (rENaC) and human ENaC (hENaC). Co-expression of rENaC and STX3 or STX4 in *Xenopus laevis* oocytes increased amiloride-sensitive whole-cell currents (Δ*I*_ami_) on average by 50% and 135%, respectively, compared to oocytes expressing rENaC alone. In contrast, STX2 had no significant effect on rENaC. Similar to its effect on rENaC, STX3 stimulated hENaC by 48%. In contrast, STX2 and STX4 inhibited hENaC by 51% and 44%, respectively. Using rENaC carrying a FLAG tag in the extracellular loop of the β-subunit, we demonstrated that the stimulatory effects of STX3 and STX4 on Δ*I*_ami_ were associated with an increased expression of the channel at the cell surface. Co-expression of STX3 or STX4 did not significantly alter the degree of proteolytic channel activation by chymotrypsin. STX3 had no effect on the inhibition of rENaC by brefeldin A, and the stimulatory effect of STX3 was preserved in the presence of dominant negative Rab11. This indicates that the stimulatory effect of STX3 is not mediated by inhibiting channel retrieval or by stimulating fusion of recycling endosomes. Our results suggest that the effects of syntaxins on ENaC are isoform and species dependent. Furthermore, our results demonstrate that STX3 increases ENaC expression at the cell surface, probably by enhancing insertion of vesicles carrying newly synthesized channels.

## Introduction

The amiloride sensitive epithelial sodium channel (ENaC) is expressed in many sodium absorbing epithelia like the aldosterone sensitive distal nephron (ASDN), distal colon, respiratory and alveolar epithelia, and the ducts of salivary and sweat glands [15, 16, 24, 50]. It mediates the critical step of passive sodium influx across the apical membrane, followed by active extrusion of sodium by the Na^+^/K^+^-ATPase across the basolateral membrane. The importance of regulating ENaC activity in principal cells of the ASDN for long-term regulation of arterial blood pressure is evidenced by two genetic diseases that affect ENaC function and arterial blood pressure: Liddle’s syndrome (pseudohyperaldosteronism) [32, 39, 67] and pseudohypoaldosteronism type 1 (PHA-1) [6]. ENaC is a member of the ENaC/DEG ion channel family [22]. It is composed of three homologous subunits (α-, β-, γ-ENaC) which most likely form a heterotrimer [40, 62]. Each subunit consists of two transmembrane domains, a large extracellular domain and intracellular N- and C-termini [16, 22]. The extracellular domains of α- and γ-ENaC are important for channel activation. Proteolytic cleavage of these domains by serine proteases is thought to release inhibitory peptides which increases channel open probability (*Po*) [14, 23, 51]. The C-terminus of each ENaC subunit is important for channel internalization. The E3 ubiquitin ligase Nedd4-2 interacts with a PY-motif present at the C-terminus of each subunit and facilitates ubiquitination of the channel which marks it for endocytosis and subsequent degradation [11, 12, 29, 36, 49, 61, 69]. The main hormonal regulator of ENaC, aldosterone, mediates its stimulatory effect on Na^+^ reabsorption in the ASDN in part via this pathway. Aldosterone increases expression of the serum and glucocorticoid-inducible kinase 1 (Sgk1) which phosphorylates Nedd4-2, thereby reducing the interaction of Nedd4-2 with the PY-motif [9, 60]. This reduces ENaC endocytosis and increases the number of ENaCs at the plasma membrane. Stimulating forward trafficking of ENaC is another way of increasing the number of channels at the plasma membrane and is likely to contribute to the stimulatory effect of aldosterone on ENaC [13]. However, in contrast to the detailed knowledge on regulation of ENaC endocytosis, knowledge on the mechanism and regulation of ENaC forward trafficking is still limited.

As a part of the SNARE (soluble *N*-ethylmaleimide sensitive factor attachment protein receptor) machinery, syntaxins are involved in membrane fusion processes. Together with SNAP(soluble *N*-ethylmaleimide sensitive factor attachment protein)-25 or SNAP-23, they form the target membrane associated t-SNARE complex, which interacts together with vesicle associated v-SNARE proteins, like synaptobrevin or other VAMPs (vesicle associated membrane proteins) to form the SNARE core complex [19, 20, 64]. The syntaxin family consists of 15 genes in mammals [64]. The first identified members, syntaxin 1A and 1B, are localized in the presynaptic plasma membrane of neuronal and secretory cells and are involved in neurotransmission and neurosecretion. In contrast, syntaxins 2, 3, and 4 are localized at the plasma membrane of various cell types and have been implicated in various processes involving the delivery of trans-Golgi network cargo to the cell surface [64]. In ENaC expressing tubular cells of rat kidney, RNA expression of syntaxin 1A and 1B is negligible, whereas RNA expression of syntaxin 4 in ASDN is comparable to that of γ-ENaC (database of nephron segment-specific transcriptomes [30]). RNA expression of syntaxin 3 is approximately a fifth of that of syntaxin 4 in cortical collecting duct (CCD) [30]. Endogenous protein expression of syntaxin 3 and syntaxin 4 has been detected in different segments of the rat nephron including the ASDN, the site of ENaC expression in kidney [3, 31, 34, 37, 38]. Conflicting data have been reported regarding the apical versus basolateral expression pattern of syntaxin 3 and syntaxin 4 in renal epithelial cells [3, 31, 37, 38]. In transfected MDCK cells, the plasma membrane localization of syntaxin 3 was found to be restricted to the apical membrane, whereas syntaxin 4 was localized basolaterally [34]. In contrast, widespread basolateral expression of syntaxin 3 [3, 38] has been reported in native rat renal epithelia. Moreover, apical [37, 38] and basolateral [31] syntaxin 4 expression has been described in rat renal epithelial cells. The latter study also reported syntaxin 2 expression at the basolateral membrane of principal cells of connecting tubule (CNT) and CCD [31]. Taken together, the presently available data suggest that syntaxin 2, syntaxin 3, and syntaxin 4 are expressed in renal epithelial cells of the ASDN. In case of an apical co-localization with ENaC, syntaxins may directly interact with the channel and modulate its function. Alternatively, they may have indirect effects on ENaC for example by affecting channel sorting and trafficking.

Asymmetric expression of transport proteins in polarized epithelial cells is an important precondition for transepithelial vectorial transport of substrates. However, it is still an enigma how polarized epithelial cells can achieve directed membrane trafficking of transport proteins to either the apical or basolateral membrane. Syntaxins are thought to play an important role in polarized membrane trafficking of transport proteins [35]. Polarized expression of syntaxins that either mediate or inhibit fusion of vesicles carrying distinct membrane proteins could favor directed delivery of vesicles and their cargo to either the apical or basolateral membrane. Interestingly, contradictory effects of syntaxins on ENaC function have been described in heterologous expression systems. For example, stimulatory and inhibitory effects of overexpressed syntaxin 1A have been reported in HT29 cells [55, 56]. Co-expression of ENaC and syntaxin 1A in *Xenopus laevis* oocytes reduced ENaC function [7, 43, 44, 54], whereas syntaxin 2 had no effect [43]. Syntaxin 3 stimulated ENaC function in one study [54] but had no effect in others [43, 44]. Some of the discrepant findings reported in the literature may be due to species differences. Surprisingly, the effect of syntaxin 4 on ENaC function has not yet been investigated. Moreover, the molecular mechanisms by which syntaxins affect ENaC function remain to be elucidated. It has been reported that syntaxin 1A interacts with cytosolic termini of ENaC [2, 7, 44, 54]. Interestingly, modifying the cytosolic termini of ENaC has been shown to affect proteolytic cleavage of extracellular regions of the α- and γ-subunit [27, 28, 52, 53]. Thus, syntaxins may alter ENaC function by modulating proteolytic ENaC activation.

The aim of the present study was to revisit some of the unresolved issues of ENaC/syntaxin interaction. To investigate their effects on ENaC function, syntaxins 2, 3, and 4 were co-expressed with rat or human ENaC in *Xenopus laevis* oocytes. Effects on ENaC-mediated whole-cell currents, channel expression at the cell surface, and on proteolytic channel activation were studied.

## Methods

### Chemicals and solutions

Unless stated otherwise, chemicals were from Sigma (Taufkirchen, Germany). Collagenase type II (CLS II) was obtained from Biochrom (Berlin, Germany). The solutions used were as follows: OR2 for isolation of oocytes (in mM: NaCl 82.5, KCl 2, MgCl_2_ 1, HEPES 5, pH 7.4 with NaOH), a low Na^+^ containing solution for oocyte incubation (in mM: NMDG-Cl 87, NaCl 9, KCl 2, CaCl_2_ 1.8, MgCl_2_ 1, HEPES 5, penicillin 100 U/ml, streptomycin 100 μg/ml, pH 7.4 with Tris), and ND96 as bath solution for two-electrode voltage-clamp experiments (in mM: NaCl 96, KCl 2, CaCl_2_ 1.8, MgCl_2_ 1, HEPES 5, pH 7.4 with Tris).

### Plasmids

Full-length cDNAs for rat α-, β-, γ-ENaC, and a rat β-ENaC mutant carrying a FLAG-epitope in the extracellular loop were in pGEM-HE and were kindly provided by Dr. Bernard Rossier and Dr. Laurent Schild (Lausanne, Switzerland). Human α-, β-, and γ-ENaC were in pcDNA3.1 and were kindly provided by Dr. Harry Cuppens and Dr. Jean-Jacques Cassiman (Leuven, Belgium). Rat syntaxins 2, 3, and 4 were kindly provided by Dr. Mike Edwardson (Cambridge, UK) and were subcloned into pTLN vector [33] which was kindly provided by Dr. Thomas Jentsch (Berlin, Germany). Human dominant-negative Rab11a was in pcDNA3.1. It carried a S25N mutation for inactivation and was kindly provided by Dr. Guiscard Seebohm (Bochum, Germany). Linearized plasmids were used as templates for cRNA synthesis (mMessage mMachine, Ambion, Austin, TX, USA) using T7 or SP6 as promotor.

### Isolation of oocytes and injection of cRNA

Defolliculated stage V-VI oocytes were obtained from ovarian lobes of adult female *Xenopus laevis* in accordance with the principles of German legislation, with approval by the animal welfare officer for the University of Erlangen-Nürnberg (FAU) and under the governance of the state veterinary health inspectorate [29, 46, 47]. Animals were anesthetized in 0.2% MS222, and ovarian lobes were excised by partial ovariectomy. Oocytes were isolated by enzymatic digestion at 19 °C for 3–4 h with 600–700 U/ml collagenase type II dissolved in OR2 solution. cRNAs were dissolved in RNase-free water and a volume of 46 nl was injected into stage V-VI oocytes with a Nanoject II automatic injector (Drummond, Broomall, PA). Amount and type of injected cRNA are specified in the figure legends. cRNAs for the three subunits (αβγ) of ENaC were routinely co-injected using 0.01, 0.025, or 0.05 ng per subunit per oocyte for rat ENaC (rENaC) and 0.2 ng per subunit per oocyte for human ENaC (hENaC). Injected oocytes were kept in a low Na^+^ containing solution to prevent Na^+^ overloading of ENaC expressing oocytes which would reduce ENaC cell surface expression [26, 65]. The incubation solution was supplemented with 100 U/ml Na^+^-penicillin and 100 μg/ml streptomycin sulfate to prevent bacterial growth.

### Two-electrode voltage-clamp

Two days after cRNA injection, whole-cell currents were measured with an OC-725C two-electrode voltage-clamp amplifier (Warner Instruments, Hamden, CT) at a holding potential of − 60 mV [29, 46, 47]. The amplifier was connected to a PC via a LIH-1600 interface (HEKA, Lambrecht, Germany) and controlled by PULSE software (HEKA) for data acquisition and analysis. During the measurements, oocytes were constantly superfused (2–3 ml/min) with ND96 at room temperature (20–21 °C). Bath solution exchanges were controlled by an ALA BPS-8 magnetic valve system in combination with a TIB14 interface (both HEKA). By convention, inward whole-cell currents evoked by influx of cations or efflux of anions are represented as negative currents. Amiloride-sensitive whole-cell current (Δ*I*_ami_) was determined by subtracting the whole-cell current measured in the absence of amiloride from that measured in the presence of amiloride (2 μM).

### Surface Labelling

ENaC surface expression was determined using a chemiluminescence assay and a rat β_FLAG_ENaC construct with a FLAG reporter epitope inserted into its extracellular domain as described previously [10, 25, 45, 48, 68]. All steps were performed on ice and no glassware was used. Unspecific binding sites were blocked by 30 min incubation in ND96 supplemented with 1% bovine serum albumin (BSA). Subsequently, oocytes were incubated for 1 h in primary mouse anti-FLAG M2 monoclonal antibody (1 μg/ml, Sigma, Taufkirchen, Germany), washed 6 × 3 min in ND96 + BSA, incubated for 45 min in secondary peroxidase-conjugated sheep anti-mouse IgG (1:400, Chemicon, Boronia Victoria, Australia), washed 6 × 3 min in ND96 + BSA, and finally 6 × 3 min in ND96. Individual oocytes were placed in a white U-bottom 96-well plate, and 50 μl of SuperSignal ELISA Femto Maximum Sensitivity Substrate (Pierce, Rockford, IL) was added to each oocyte. Chemiluminescence was quantified with a Tecan GENios microplate reader (TECAN, Crailsheim, Germany).

### Statistics

Data are presented as mean ± SEM and were analyzed using GraphPad Prism 5.04 for Windows (GraphPad Software Inc., San Diego, CA, USA). Statistical significance was assessed by appropriate versions of Student’s *t* test or ANOVA. Figures in columns indicate number of measured oocytes; *N* indicates the number of different batches of oocytes.

## Results

### Syntaxins 3 and 4 stimulate rENaC but syntaxin 2 does not

**Fig. 1:**
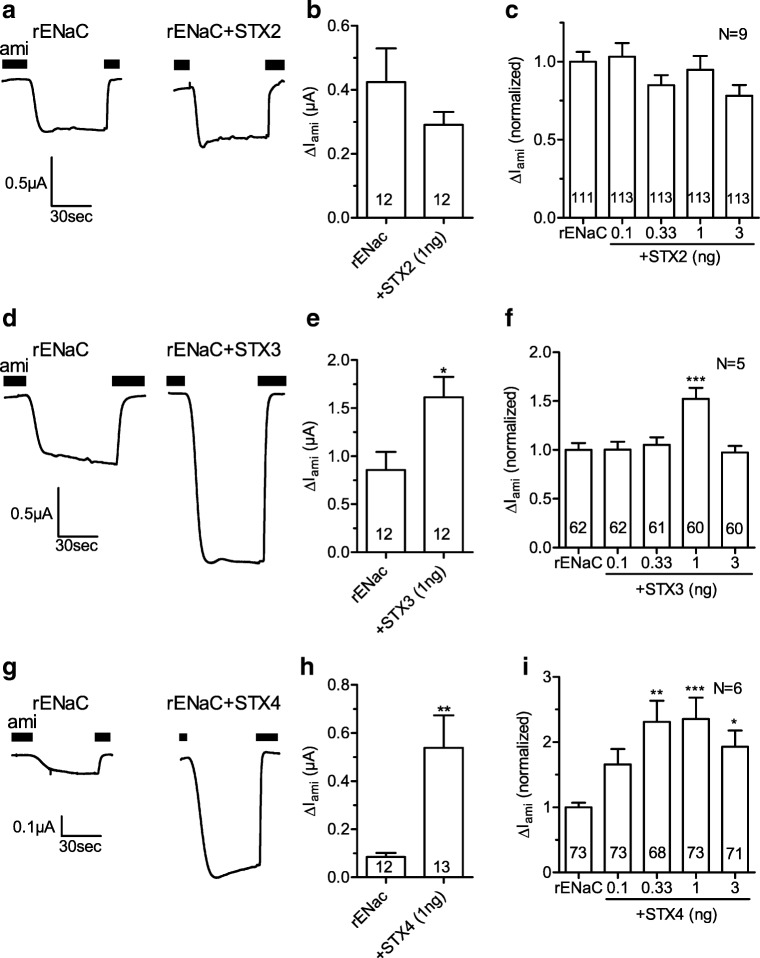
Syntaxins 3 and 4 stimulate rENaC function in oocytes. Oocytes were injected with cRNA for rENaC alone or together with different amounts of cRNA for syntaxin 2 (+STX2), syntaxin 3 (+STX3), or syntaxin 4 (+STX4) and incubated for 2 days. Amiloride-sensitive whole-cell currents (Δ*I*_ami_) were measured with the two-electrode voltage-clamp technique. **a**, **d**, **g** Representative whole-cell current traces from matched control oocytes expressing rENaC alone or from oocytes co-expressing rENaC and a syntaxin isoform (1 ng cRNA), as indicated. The presence of amiloride (ami) in the bath solution is indicated by black bars. **b**, **e**, **h** Summary of data obtained from an individual batch of oocytes measured as shown in **a**, **d**, or **g**, respectively. **c**, **f**, **i** Summary of data as shown in **b**, **e**, and **h** obtained from several different batches of oocytes. To take batch-to-batch variability into account, individual Δ*I*_ami_ values were normalized to the mean Δ*I*_ami_ of the rENaC control group of the corresponding batch. Unpaired *t* test (**b**, **e**, **h**) or one-way ANOVA followed by Dunnett’s multiple comparison test vs. rENaC control (**c**, **f**, **i**), * *p* < 0.05, ** *p* < 0.01, *** *p* < 0.001

To investigate the effects of syntaxins 2, 3, and 4 on rat ENaC function, *Xenopus laevis* oocytes were injected with cRNA for rENaC alone or in combination with different amounts of cRNA for syntaxins 2, 3, or 4. After 2 days of incubation, amiloride-sensitive whole-cell currents (Δ*I*_ami_) were measured with the two-electrode voltage-clamp technique. Figure 1a shows representative whole-cell current traces from an oocyte expressing rENaC alone and from an oocyte co-expressing rENaC and syntaxin 2 (1 ng cRNA). Average data from similar experiments using oocytes from the same batch are shown in Fig. 1b. Normalized data from different batches of oocytes using different amounts of injected cRNA for syntaxin 2 are summarized in Fig. 1c. Taken together, the experiments shown in Fig. 1a–c revealed no significant effect of syntaxin 2 co-expression on ENaC currents which is in agreement with a previous report [43]. In contrast to syntaxin 2, co-expression of syntaxin 3 (1 ng cRNA) stimulated Δ*I*_ami_ (Fig. 1d, e). Interestingly, a significant stimulatory effect of syntaxin 3 was only observed in experiments with 1 ng of injected cRNA for syntaxin 3 but not with lower or higher amounts (Fig. 1f). On average, co-injection of 1 ng of cRNA for syntaxin 3 increased rENaC currents by 50% compared to Δ*I*_ami_ measured in corresponding control oocytes injected with cRNA for rENaC alone. Co-injection of cRNA for syntaxin 4 (0.33, 1, or 3 ng) also significantly increased Δ*I*_ami_ (Fig. 1g–i) with a maximal average stimulation of 135% observed with 1 ng of cRNA.

### hENaC is stimulated by syntaxin 3 but not by syntaxins 2 and 4

**Fig. 2:**
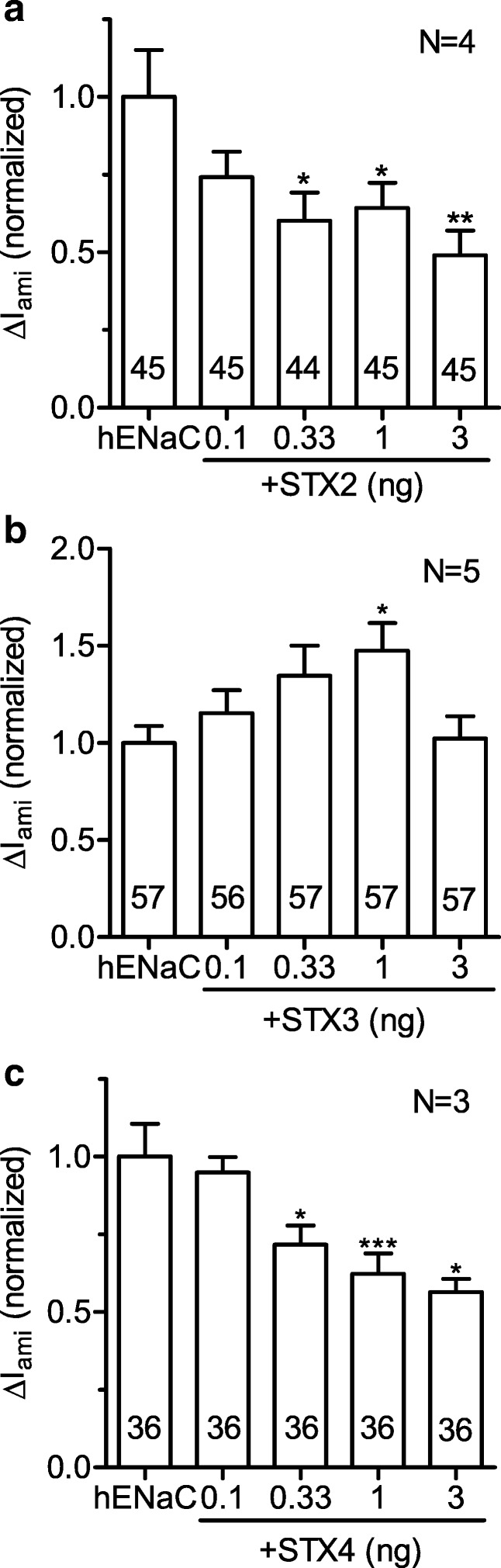
Effects of syntaxins 2, 3, and 4 on hENaC function. Oocytes were injected with cRNA for hENaC alone or together with different amounts of cRNA for syntaxin 2 (+STX2, **a**), syntaxin 3 (+STX3, **b**), or syntaxin 4 (+STX4, **c**). To take batch-to-batch variability into account, individual Δ*I*_ami_ values were normalized to the mean Δ*I*_ami_ of the hENaC control group of the corresponding batch. One-way ANOVA followed by Dunnett’s multiple comparison test vs. hENaC control, *p < 0.05, **p < 0.01, ***p < 0.001

To investigate whether syntaxins also affect human ENaC, we measured whole-cell currents from oocytes expressing hENaC alone or co-expressing hENaC and syntaxin 2, 3, or 4. As summarized in Fig. 2b, co-expression of syntaxin 3 (1 ng cRNA) stimulated human ENaC to a similar extent (48%) as rat ENaC. There was a non-significant stimulatory trend with 0.1 and 0.33 ng cRNA for syntaxin 3, but no appreciable effect with 3 ng. Interestingly, co-injection of cRNA for syntaxin 2 (0.33, 1, or 3 ng; Fig. 2a) or syntaxin 4 (0.33, 1, or 3 ng; Fig. 2c) had a significant inhibitory effect on hENaC. The inhibitory effect was most pronounced with 3 ng cRNA with a 51% inhibition by syntaxin 2 and a 44% inhibition by syntaxin 4. The inhibitory effects of syntaxin 2 and syntaxin 4 on hENaC differ from the findings with rENaC shown above. Thus, effects of different syntaxin isoforms on ENaC may be species dependent. This may explain some of the controversial findings reported in the literature. The preserved stimulatory effect of syntaxin 3 on human ENaC makes syntaxin 3 a particularly interesting candidate for further studies.

### Syntaxins 3 and 4 increase rENaC expression at the cell surface

**Fig. 3:**
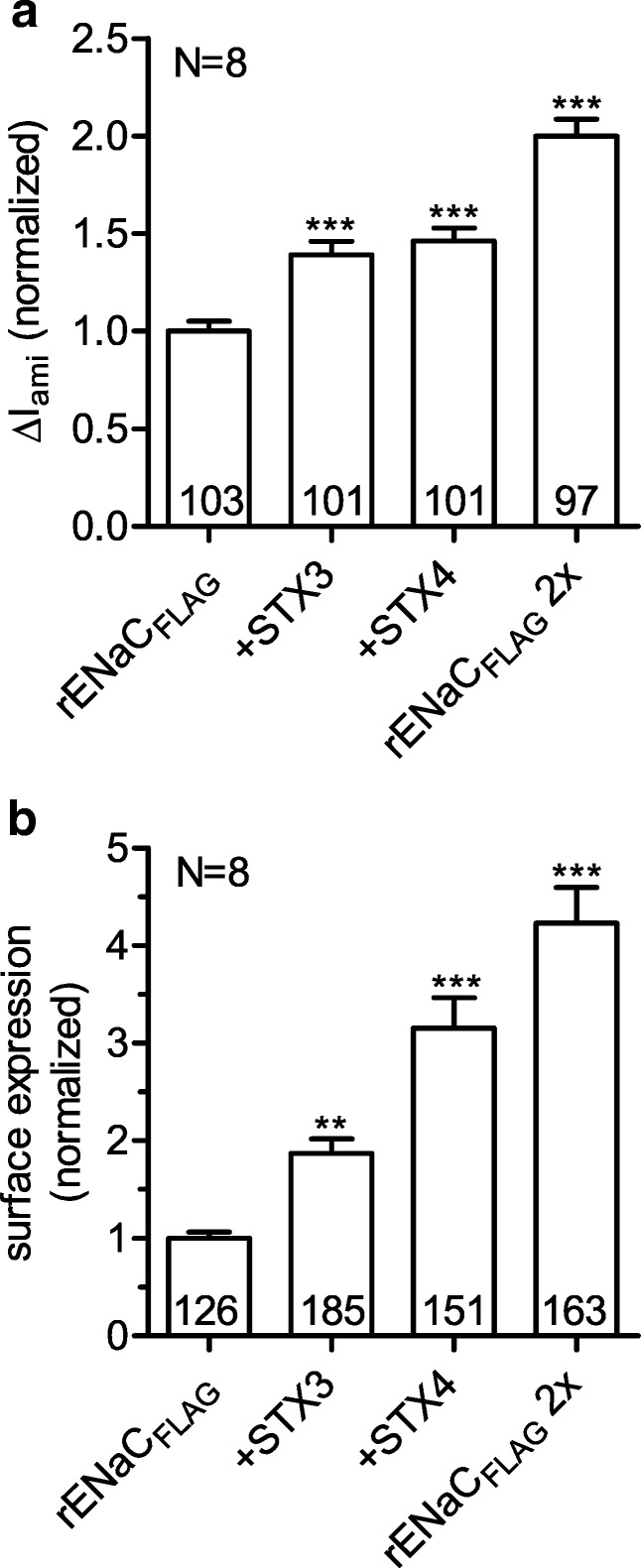
Syntaxins 3 and 4 increase surface expression of rENaC. Δ*I*_ami_ (**a**) and surface expression (**b**) were measured in parallel in oocytes expressing rENaC carrying a FLAG reporter epitope in the extracellular domain of the β-subunit (rENaC_FLAG_) alone or together with syntaxin 3 (+STX3) or syntaxin 4 (+STX4). Oocytes injected with twice the amount of cRNA for rENaC_FLAG_ (rENaC_FLAG_ 2×) served as positive control. In three batches of oocytes, oocytes were injected with 0.025 ng cRNA/subunit for rENaC_FLAG_ and with 1 ng cRNA for syntaxin 3 or syntaxin 4. In five batches of oocytes, oocytes were injected with 0.05 ng cRNA/subunit for rENaC_FLAG_ and with 2 ng cRNA for syntaxin 3 or syntaxin 4. To summarize data from different batches of oocytes, values were normalized to the mean of the corresponding rENaC_FLAG_ control group. One-way ANOVA followed by Dunnett’s multiple comparison test vs. rENaC_FLAG_ control, **p < 0.01, ***p < 0.001

Syntaxins are involved in vesicle fusion processes. Therefore, we hypothesized that the stimulatory effect of syntaxin 3 and syntaxin 4 on rENaC function is caused by an increase of channel expression at the cell surface. To investigate this hypothesis, we detected rENaC expression at the cell surface of rENaC expressing oocytes with a chemiluminescence based assay in the presence and absence of syntaxin 3 or syntaxin 4. For channel detection at the cell surface, a FLAG reporter epitope was inserted in the extracellular domain of the β-rENaC subunit. As positive control for increased channel surface expression, an additional group of oocytes was injected with twice the amount of cRNA for rENaC and was studied in parallel in every set of experiments. Δ*I*_ami_ was measured in matched groups of oocytes from the same batches as used for the detection of rENaC surface expression. Doubling the amount of injected cRNA for rENaC increased Δ*I*_ami_ by about 2-fold and the corresponding chemiluminescence signal by about 4-fold (Fig. 3). Thus, the relative increase in chemiluminescence is larger than that of the corresponding ENaC currents. Nevertheless, these control experiments confirmed the suitability of the chemiluminescence assay to detect an increase in ENaC surface expression in a semiquantitative manner. Co-expression of rENaC and syntaxin 3 increased Δ*I*_ami_ 1.4-fold and surface expression 1.9-fold compared to oocytes expressing rENaC alone. Similarly, syntaxin 4 increased Δ*I*_ami_ and surface expression 1.5-fold and 3.2-fold, respectively. Taken together, these data suggest that the stimulatory effects of syntaxin 3 and syntaxin 4 can be attributed at least in part, if not fully, to an increase of rENaC surface expression.

### Syntaxins 3 and 4 do not affect proteolytic activation of rENaC by chymotrypsin

**Fig. 4:**
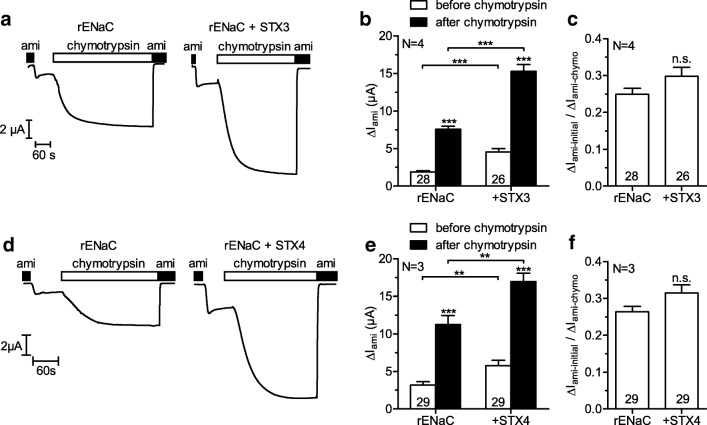
Syntaxins 3 and 4 do not affect proteolytic activation of rENaC by chymotrypsin. Oocytes were injected with cRNA for rENaC (0.025 ng/subunit) alone or together with cRNA for syntaxin 3 (STX3, 1 ng) or syntaxin 4 (STX4, 1 ng) and incubated for 2 days. **a**, **d** Typical whole-cell current traces of oocytes expressing rENaC alone or together with syntaxin 3 (**a**) or syntaxin 4 (**d**). **b**, **e** Summary of Δ*I*_ami_ values before and after activation of rENaC with chymotrypsin (2 μg/ml) obtained from similar experiments as shown in **a** and **d**, respectively. Δ*I*_ami_ values before and after application of chymotrypsin were compared with paired *t* test; corresponding Δ*I*_ami_ values in the absence and presence of syntaxin 3 or syntaxin 4 were compared with unpaired *t* test. **c**, **f** Ratio of Δ*I*_ami_ before (Δ*I*_ami-initial_) and after (Δ*I*_ami-chymo_) activation of rENaC with chymotrypsin was calculated from individual oocytes as a measure of average channel *Po*; unpaired *t* test. *n.s.* not significant, **p < 0.01, ***p < 0.001

To investigate a possible effect of syntaxin 3 and syntaxin 4 on proteolytic ENaC activation, we expressed rENaC alone or together with syntaxin 3 or syntaxin 4 in oocytes and measured Δ*I*_ami_ before and after proteolytic activation of ENaC with chymotrypsin, which maximizes channel *Po* [17, 46, 47]. After maximizing *Po*, differences in Δ*I*_ami_ are largely independent of *Po* and therefore reflect differences of channel surface expression. In addition, the ratio of Δ*I*_ami_ before and after rENaC activation is a good estimate of average rENaC *Po* prior to activation. Figure 4a and d show representative whole-cell current traces from individual oocytes to illustrate the experimental protocol and key findings. All experiments were started in the presence of amiloride. Δ*I*_ami-initial_ was determined from the new steady state whole-cell current reached after washout of amiloride. Subsequently, chymotrypsin was added to the bath solution. The increase of the inward whole-cell current reflects proteolytic channel activation. After the whole-cell current had reached a new steady state, amiloride was re-applied and Δ*I*_ami-chymo_ was determined. As expected, baseline ENaC currents (Δ*I*_ami-initial_) were larger in oocytes co-expressing syntaxin 3 (Fig. 4a, b) or syntaxin 4 (Fig. 4d, e) compared to the corresponding control oocytes. Thus, these experiments confirmed the stimulatory effect of syntaxin 3 and syntaxin 4 on ENaC shown above. Consistent with the increased baseline ENaC currents, application of chymotrypsin increased ENaC currents to higher absolute values in oocytes co-expressing syntaxin 3 or syntaxin 4 compared to those reached in the corresponding control oocytes expressing rENaC alone (Fig. 4b, e). Importantly, the relative stimulatory effect of chymotrypsin on ENaC currents in control oocytes was not significantly different from that in oocytes co-expressing syntaxin 3 (4.7-fold vs. 4.1-fold) or syntaxin 4 (4.2-fold vs. 3.6-fold). Moreover, neither syntaxin 3, nor syntaxin 4 showed significant effects on the ratio of Δ*I*_ami-initial_ and Δ*I*_ami-chymo_ (Fig. 4c, f) which suggests that the effects of syntaxin 3 and syntaxin 4 on rENaC *Po* are negligible. In summary, these results confirm the conclusion from the chemiluminescence experiments (see above) and support the hypothesis that syntaxin 3 and syntaxin 4 stimulated rENaC function mainly by increasing channel surface expression without a major effect on *Po*.

### Syntaxin 3 stimulates rENaC insertion into the plasma membrane

**Fig. 5:**
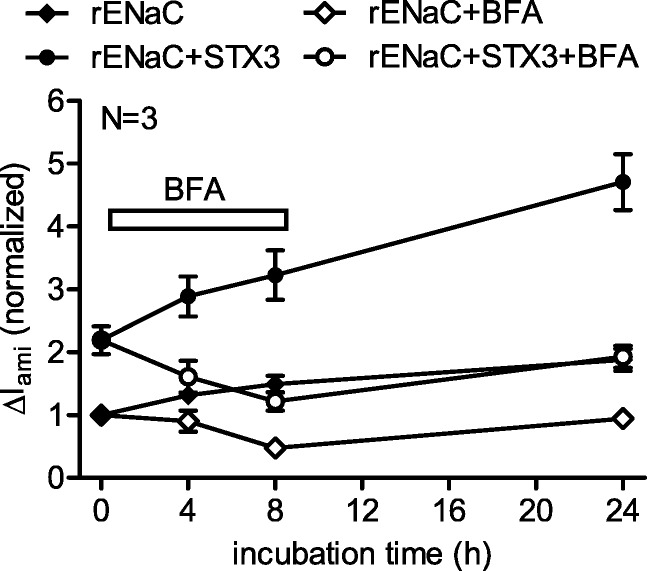
Syntaxin 3 stimulates rENaC insertion into the plasma membrane. Oocytes were injected with cRNA for rENaC (0.025 ng/subunit) alone or together with cRNA for syntaxin 3 (1 ng) and incubated for 2 days. After the initial measurement of Δ*I*_ami_ at 0 h, half of the oocytes were incubated for 8 h in the presence of brefeldin A (BFA, 5 μM), as indicated, and Δ*I*_ami_ was measured at 4, 8, and 24 h. Each data point represents Δ*I*_ami_ values from 28 to 30 oocytes. For better comparison, Δ*I*_ami_ was normalized to the rENaC expressing control group

The stimulatory effect of syntaxin 3 on rENaC surface expression can be caused by a stimulation of channel insertion into the plasma membrane or by an inhibition of channel retrieval. To investigate by which of these mechanisms syntaxin 3 stimulates channel surface expression, we blocked channel delivery from the Golgi apparatus to the plasma membrane by brefeldin A [42, 59]. Blocking channel delivery to the plasma membrane decreases rENaC surface expression and thus Δ*I*_ami_. If syntaxin 3 stimulated channel insertion into the plasma membrane, it is not expected to affect the inhibitory effect of brefeldin A on Δ*I*_ami_. In contrast, if syntaxin 3 inhibited channel retrieval, it is likely to diminish the inhibitory effect of brefeldin A. Co-expression of syntaxin 3 stimulated Δ*I*_ami_ at 0 h baseline by 119% (Fig. 5), which confirms the stimulatory effect of syntaxin 3 on rENaC in the experiments performed with brefeldin A. Incubating the oocytes in the presence of brefeldin A reduced Δ*I*_ami_ in oocytes with and without co-expression of syntaxin 3 consistent with an inhibition of anterograde channel trafficking. In contrast, in time-matched control oocytes incubated in the absence of brefeldin A, Δ*I*_ami_ continuously increased over 8 h indicating ongoing channel insertion. Importantly, in oocytes expressing rENaC alone or rENaC and syntaxin 3, brefeldin A reduced Δ*I*_ami_ by 68% and 62%, respectively, compared to corresponding control oocytes incubated in the absence of brefeldin A. Thus, the inhibitory effect of brefeldin A was preserved in oocytes co-expressing syntaxin 3 which argues against an effect of syntaxin 3 on channel retrieval. After brefeldin A was removed from the incubation medium, Δ*I*_ami_ increased in all experimental groups at a similar rate which confirmed that the oocytes remained viable. In summary, these results suggest that syntaxin 3 stimulates rENaC insertion into the plasma membrane.

### Blocking endosomal recycling does not prevent the stimulation of rENaC by syntaxin 3

**Fig. 6:**
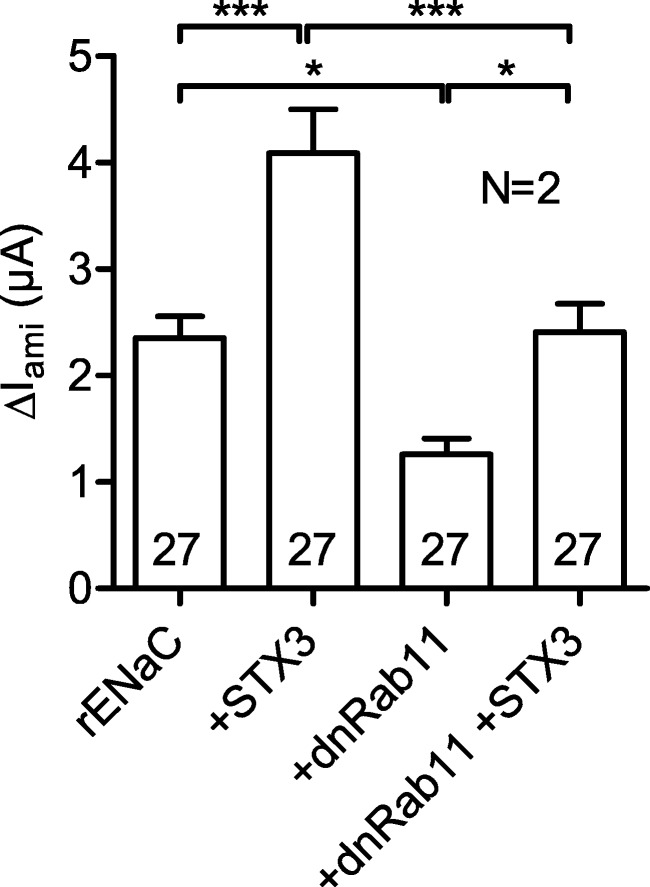
Blocking endosomal recycling does not prevent the stimulation of rENaC by syntaxin 3. Oocytes were injected with cRNA for rENaC (0.025 ng/subunit) alone or together with cRNA for dominant-negative Rab11a (5 ng, +dnRab11a), syntaxin 3 (1 ng, +STX3), or both. Δ*I*_ami_ was measured after 2 days of incubation. One-way ANOVA followed by Bonferroni’s multiple comparison test with selected pairs, *p < 0.05, **p < 0.01, ***p < 0.001

rENaC expression at the plasma membrane can be increased by stimulating fusion of vesicles carrying newly synthesized channels from the Golgi apparatus or by recycling endosomes to the plasma membrane which results in the reinsertion of previously endocytosed rENaC. The small G protein Rab11a has been shown to be involved in the regulation of ENaC trafficking by endosomal recycling [5, 21]. If the stimulatory effect of syntaxin 3 on rENaC is mediated by increased endosomal recycling, blocking this pathway is expected to reduce the stimulatory effect of syntaxin 3. To test this hypothesis, we expressed rENaC alone or together with syntaxin 3 and/or dominant-negative Rab11a (dnRab11a) in oocytes. Compared to rENaC expressing control oocytes, co-expression of syntaxin 3 stimulated Δ*I*_ami_ by 74% which confirms the stimulatory effect of syntaxin 3 in this set of experiments. Co-expression of rENaC and dnRab11a reduced Δ*I*_ami_ by 47% (Fig. 6) which indicates the inhibitory effect of dnRab11a on endosomal recycling. Co-expression of rENaC, dnRab11a, and syntaxin 3 increased Δ*I*_ami_ by 91% compared to oocytes co-expressing rENaC and dnRab11a. Thus, blocking endosomal recycling by dnRab11a did not prevent the stimulatory effect of syntaxin 3. These results support the conclusion that syntaxin 3 increases rENaC expression at the cell surface probably by enhancing the insertion of vesicles carrying newly synthesized channels.

## Discussion

In the present study, we report for the first time that syntaxin 4 stimulates rENaC function. The stimulatory effect could be attributed to an increase of rENaC surface expression, whereas average *Po* of rENaC was not altered by syntaxin 4. In contrast to its effect on rENaC, syntaxin 4 inhibited hENaC. It is tempting to speculate that subtle species differences may be responsible for the opposite effects of rat syntaxin 4 on rENaC and hENaC. Importantly, the observed opposite effects argue against a nonspecific action of syntaxin 4 in the oocyte expression system, because this would be expected to affect rENaC and hENaC in a similar manner.

Syntaxin 3 consistently stimulated rENaC and hENaC in the present study. This is an observation with potential physiological relevance, because syntaxin 3 may be co-expressed with ENaC in the apical membrane of renal tubular cells in the ASDN. This stimulatory effect of syntaxin 3 was consistently observed in experiments with 1 ng of injected syntaxin 3 cRNA. Interestingly, with a higher amount of cRNA, the stimulatory effect was not enhanced but disappeared. A similar phenomenon has previously been reported for syntaxin 1A in human HT29 colonic epithelial cells, which endogenously express ENaC [56]. The authors speculated that higher expression of syntaxin 1A may have reduced stimulation as a consequence of overdose. Interestingly, the N-terminus of some syntaxins is able to reversibly associate with their own H3 domain which prevents the formation of the SNARE core complex [20, 64]. It is conceivable that, as an unspecific effect, the N-terminus of overexpressed syntaxins may interact with the H3 domains of endogenous syntaxins or of other overexpressed syntaxins, thereby inhibiting the formation of the SNARE core complex. This could explain the overdose effect. In this context, it is interesting to mention that overexpression of syntaxin 1A in HT29 cells stimulated endogenous ENaC, whereas overexpression of isolated H3 domains of syntaxin 1A inhibited ENaC [56].

The stimulatory effect of syntaxin 3 on rENaC seen in the present study can be attributed to an increase of rENaC surface expression, whereas average *Po* of rENaC does not seem to be altered by syntaxin 3. In agreement with our data, a previous study performed in oocytes also reported an increase of rENaC surface expression induced by syntaxin 3 [54]. In contrast, another study found no effect of syntaxin 3 on hENaC surface expression and Δ*I*_ami_ [44]. We do not know the reason for this discrepancy. However, considering the dose dependent effect seen in the present study with different amounts of injected cRNA for syntaxin 3, the effective dose may have been missed in the latter study.

An increase of channel expression at the cell surface can be the result of an increased fusion of trafficking vesicles with the plasma membrane or of an inhibition of channel retrieval. To distinguish between these mechanisms, we incubated oocytes expressing rENaC alone or together with syntaxin 3 in the presence of brefeldin A, a fungal metabolite which blocks channel delivery from the Golgi apparatus to the plasma membrane [42]. This method has previously been used in several studies with *Xenopus laevis* oocytes [1, 26, 41, 44, 59, 65]. In the present study, the inhibitory effect of brefeldin A on Δ*I*_ami_ was preserved in the presence of syntaxin 3. This result suggests that syntaxin 3 does not inhibit channel retrieval but stimulates channel insertion. Delivery of ENaC to the plasma membrane may be directly from the Golgi apparatus, from the recycling pathway or from stored vesicles [4, 66]. The relative importance of these delivery pathways is likely to be regulated according to physiological needs and probably varies in different tissues and cellular expression systems. For example, internalized ENaC showed poor recycling in MDCK cells under baseline conditions, whereas ENaC recycling was strongly enhanced by long-term stimulation with cAMP [36]. Effects of Rab11a and Rab11b on ENaC activity indicate that recycling endosomes play a role in ENaC delivery to the plasma membrane [4, 66]. Rab11a has been shown to increase ENaC activity in CHO cells [21], and a dominant negative form of Rab11a has been shown to inhibit ENaC-dependent sodium transport in mpkCCD cells [5]. To exclude that in the oocyte expression system syntaxin 3 stimulates insertion of recycling endosomes, we blocked insertion of recycling endosomes with dominant negative Rab11a. Rab11a is a small G protein (GTPase) which is expressed at the membranes of the Golgi apparatus, early endosomes, and recycling endosomes and regulates slow endocytic recycling [18]. The dominant negative Rab11a mutant has previously been used in *Xenopus laevis* oocytes to prevent stimulation of KCNQ1/KCNE1 potassium channels, the AMPA-type glutamate receptor GluA1 and a kainate receptor mutant (GluR6-M836I) by blocking endosomal recycling [57, 58, 63]. In the present study, dnRab11a reduced the function of rENaC which confirmed its inhibitory effect on endosomal recycling. Importantly, the stimulatory effect of syntaxin 3 was preserved in the presence of dnRab11a. This supports the conclusion that syntaxin 3 enhances ENaC surface expression by favoring fusion of vesicles carrying newly synthesized ENaCs with the plasma membrane and not by stimulating endosomal recycling. It should be noted that trafficking of membrane proteins in *Xenopus laevis* oocytes may differ from that in polarized epithelial cells, which is a limitation of the present study.

Serine proteases, e.g., chymotrypsin, are able to stimulate ENaC by increasing its *Po* [14, 23, 51]. In the present study, neither syntaxin 3 nor syntaxin 4 affected proteolytic activation of rENaC by chymotrypsin. These results suggest that average *Po* of rENaC was not affected by syntaxin 3 or syntaxin 4. Previously, it has been reported that GST-syntaxin 1A fusion proteins including the H3 domain reduced ENaC *Po* in single-channel recordings from planar lipid bilayers and from *Xenopus laevis* oocytes in the cell-attached mode [2, 8]. Thus, the effects of syntaxin 3 and syntaxin 4 on ENaC apparently differ from that of syntaxin 1A regarding *Po*.

In summary, we have shown that syntaxin 3 and syntaxin 4 consistently stimulated rENaC function mainly by increasing channel surface expression with little effect on *Po*. Moreover, we provide evidence that syntaxin 3 stimulates rENaC by increasing the delivery of newly synthesized ENaC to the plasma membrane but not by stimulating endosomal recycling. The stimulatory effect of syntaxin 3 was confirmed for hENaC, but in contrast to its stimulatory effect on rENaC, syntaxin 4 had an inhibitory effect on hENaC. Moreover, syntaxin 2 had no detectable effect on rENaC but an inhibitory effect on hENaC. These latter findings support the hypothesis that species differences may explain some of the controversial findings reported on ENaC/syntaxin interaction in the literature. The observed stimulatory effect of syntaxin 3 may be physiologically relevant for ENaC regulation in the apical membrane of tubular cells in the ASDN where ENaC and syntaxin 3 may be colocalized.

## References

[1] Anantharam A, Tian Y, Palmer LG (2006). Open probability of the epithelial sodium channel is regulated by intracellular sodium. J Physiol.

[2] Berdiev BK, Jovov B, Tucker WC, Naren AP, Fuller CM, Chapman ER, Benos DJ (2004). ENaC subunit-subunit interactions and inhibition by syntaxin 1A. Am J Physiol Renal Physiol.

[3] Breton S, Inoue T, Knepper MA, Brown D (2002). Antigen retrieval reveals widespread basolateral expression of syntaxin 3 in renal epithelia. Am J Physiol Renal Physiol.

[4] Butterworth MB (2010). Regulation of the epithelial sodium channel (ENaC) by membrane trafficking. Biochim Biophys Acta.

[5] Butterworth MB, Edinger RS, Silvis MR, Gallo LI, Liang X, Apodaca G, Frizzell RA, Johnson JP (2012). Rab11b regulates the trafficking and recycling of the epithelial sodium channel (ENaC). Am J Physiol Renal Physiol.

[6] Chang SS, Grunder S, Hanukoglu A, Rosler A, Mathew PM, Hanukoglu I, Schild L, Lu Y, Shimkets RA, Nelson-Williams C, Rossier BC, Lifton RP (1996). Mutations in subunits of the epithelial sodium channel cause salt wasting with hyperkalaemic acidosis, pseudohypoaldosteronism type 1. Nat Genet.

[7] Condliffe SB, Carattino MD, Frizzell RA, Zhang H (2003). Syntaxin 1A regulates ENaC via domain-specific interactions. J Biol Chem.

[8] Condliffe SB, Zhang H, Frizzell RA (2004). Syntaxin 1A regulates ENaC channel activity. J Biol Chem.

[9] Debonneville C, Flores SY, Kamynina E, Plant PJ, Tauxe C, Thomas MA, Munster C, Chraibi A, Pratt JH, Horisberger JD, Pearce D, Loffing J, Staub O (2001). Phosphorylation of Nedd4-2 by Sgk1 regulates epithelial Na^+^ channel cell surface expression. EMBO J.

[10] Firsov D, Schild L, Gautschi I, Merillat AM, Schneeberger E, Rossier BC (1996). Cell surface expression of the epithelial Na channel and a mutant causing Liddle syndrome: a quantitative approach. Proc Natl Acad Sci U S A.

[11] Foot N, Henshall T, Kumar S (2017). Ubiquitination and the regulation of membrane proteins. Physiol Rev.

[12] Fotia AB, Dinudom A, Shearwin KE, Koch JP, Korbmacher C, Cook DI, Kumar S (2003). The role of individual Nedd4-2 (KIAA0439) WW domains in binding and regulating epithelial sodium channels. FASEB J.

[13] Frindt G, Bertog M, Korbmacher C, Palmer LG (2020) Ubiquitination of renal ENaC subunits in vivo. Am J Physiol Renal Physiol. 10.1152/ajprenal.00609.2019 10.1152/ajprenal.00609.2019 PMC7294337 32174140

[14] Gaillard EA, Kota P, Gentzsch M, Dokholyan NV, Stutts MJ, Tarran R (2010). Regulation of the epithelial Na^+^ channel and airway surface liquid volume by serine proteases. Pflugers Arch.

[15] Garty H, Palmer LG (1997). Epithelial sodium channels: function, structure, and regulation. Physiol Rev.

[16] Hanukoglu I, Hanukoglu A (2016). Epithelial sodium channel (ENaC) family: phylogeny, structure-function, tissue distribution, and associated inherited diseases. Gene.

[17] Huber R, Krueger B, Diakov A, Korbmacher J, Haerteis S, Einsiedel J, Gmeiner P, Azad AK, Cuppens H, Cassiman JJ, Korbmacher C, Rauh R (2010). Functional characterization of a partial loss-of-function mutation of the epithelial sodium channel (ENaC) associated with atypical cystic fibrosis. Cell Physiol Biochem.

[18] Hutagalung AH, Novick PJ (2011). Role of Rab GTPases in membrane traffic and cell physiology. Physiol Rev.

[19] Jahn R, Fasshauer D (2012). Molecular machines governing exocytosis of synaptic vesicles. Nature.

[20] Jahn R, Scheller RH (2006). SNAREs--engines for membrane fusion. Nat Rev Mol Cell Biol.

[21] Karpushev AV, Levchenko V, Pavlov TS, Lam V, Vinnakota KC, Vandewalle A, Wakatsuki T, Staruschenko A (2008). Regulation of ENaC expression at the cell surface by Rab11. Biochem Biophys Res Commun.

[22] Kellenberger S, Schild L (2015). International Union of Basic and Clinical Pharmacology. XCI. Structure, function, and pharmacology of acid-sensing ion channels and the epithelial Na^+^ channel. Pharmacol Rev.

[23] Kleyman TR, Carattino MD, Hughey RP (2009). ENaC at the cutting edge: regulation of epithelial sodium channels by proteases. J Biol Chem.

[24] Kleyman TR, Kashlan OB, Hughey RP (2018). Epithelial Na^+^ channel regulation by extracellular and intracellular factors. Annu Rev Physiol.

[25] Konstas AA, Bielfeld-Ackermann A, Korbmacher C (2001) Sulfonylurea receptors inhibit the epithelial sodium channel (ENaC) by reducing surface expression. Pflugers Archiv 442:752–761. 10.1007/s004240100597 10.1007/s004240100597 11512032

[26] Konstas AA, Shearwin-Whyatt LM, Fotia AB, Degger B, Riccardi D, Cook DI, Korbmacher C, Kumar S (2002). Regulation of the epithelial sodium channel by N4WBP5A, a novel Nedd4/Nedd4-2-interacting protein. J Biol Chem.

[27] Kota P, Buchner G, Chakraborty H, Dang YL, He H, Garcia GJ, Kubelka J, Gentzsch M, Stutts MJ, Dokholyan NV (2014). The N-terminal domain allosterically regulates cleavage and activation of the epithelial sodium channel. J Biol Chem.

[28] Kota P, Gentzsch M, Dang YL, Boucher RC, Stutts MJ (2018). The N terminus of α-ENaC mediates ENaC cleavage and activation by furin. J Gen Physiol.

[29] Krueger B, Yang L, Korbmacher C, Rauh R (2018). The phosphorylation site T613 in the β-subunit of rat epithelial Na^+^ channel (ENaC) modulates channel inhibition by Nedd4-2. Pflugers Arch.

[30] Lee JW, Chou CL, Knepper MA (2015). Deep sequencing in microdissected renal tubules identifies nephron segment-specific transcriptomes. J Am Soc Nephrol.

[31] Li X, Low SH, Miura M, Weimbs T (2002). SNARE expression and localization in renal epithelial cells suggest mechanism for variability of trafficking phenotypes. Am J Physiol Renal Physiol.

[32] Liddle GW, Bledsoe T, Coppage WSJ (1963). A familial renal disorder simulating primary aldosteronism but with negligible aldosterone secretion. Trans Assoc Am Phys.

[33] Lorenz C, Pusch M, Jentsch TJ (1996). Heteromultimeric CLC chloride channels with novel properties. Proc Natl Acad Sci U S A.

[34] Low SH, Chapin SJ, Weimbs T, Komuves LG, Bennett MK, Mostov KE (1996). Differential localization of syntaxin isoforms in polarized Madin-Darby canine kidney cells. Mol Biol Cell.

[35] Low SH, Chapin SJ, Wimmer C, Whiteheart SW, Komuves LG, Mostov KE, Weimbs T (1998). The SNARE machinery is involved in apical plasma membrane trafficking in MDCK cells. J Cell Biol.

[36] Lu C, Pribanic S, Debonneville A, Jiang C, Rotin D (2007). The PY motif of ENaC, mutated in Liddle syndrome, regulates channel internalization, sorting and mobilization from subapical pool. Traffic.

[37] Mandon B, Chou CL, Nielsen S, Knepper MA (1996). Syntaxin-4 is localized to the apical plasma membrane of rat renal collecting duct cells: possible role in aquaporin-2 trafficking. J Clin Invest.

[38] Mandon B, Nielsen S, Kishore BK, Knepper MA (1997). Expression of syntaxins in rat kidney. Am J Phys.

[39] Nesterov V, Krueger B, Bertog M, Dahlmann A, Palmisano R, Korbmacher C (2016). In Liddle syndrome, epithelial sodium channel is hyperactive mainly in the early part of the aldosterone-sensitive distal nephron. Hypertension.

[40] Noreng S, Bharadwaj A, Posert R, Yoshioka C, Baconguis I (2018). Structure of the human epithelial sodium channel by cryo-electron microscopy. Elife.

[41] Patel AB, Yang L, Deng S, Palmer LG (2014). Feedback inhibition of ENaC: acute and chronic mechanisms. Channels (Austin).

[42] Pelham HR (1991). Multiple targets for brefeldin A. Cell.

[43] Peters KW, Qi J, Johnson JP, Watkins SC, Frizzell RA (2001). Role of snare proteins in CFTR and ENaC trafficking. Pflugers Arch.

[44] Qi J, Peters KW, Liu C, Wang JM, Edinger RS, Johnson JP, Watkins SC, Frizzell RA (1999). Regulation of the amiloride-sensitive epithelial sodium channel by syntaxin 1A. J Biol Chem.

[45] Rauh R, Dinudom A, Fotia AB, Paulides M, Kumar S, Korbmacher C, Cook DI (2006). Stimulation of the epithelial sodium channel (ENaC) by the serum- and glucocorticoid-inducible kinase (Sgk) involves the PY motifs of the channel but is independent of sodium feedback inhibition. Pflugers Arch.

[46] Rauh R, Diakov A, Tzschoppe A, Korbmacher J, Azad AK, Cuppens H, Cassiman JJ, Dotsch J, Sticht H, Korbmacher C (2010). A mutation of the epithelial sodium channel associated with atypical cystic fibrosis increases channel open probability and reduces Na^+^ self inhibition. J Physiol.

[47] Rauh R, Soell D, Haerteis S, Diakov A, Nesterov V, Krueger B, Sticht H, Korbmacher C (2013). A mutation in the β-subunit of ENaC identified in a patient with cystic fibrosis-like symptoms has a gain-of-function effect. Am J Physiol Lung Cell Mol Physiol.

[48] Rauh R, Hoerner C, Korbmacher C (2017). δβγ-ENaC is inhibited by CFTR but stimulated by cAMP in Xenopus laevis oocytes. Am J Physiol Lung Cell Mol Physiol.

[49] Ronzaud C, Staub O (2014). Ubiquitylation and control of renal Na^+^ balance and blood pressure. Physiology.

[50] Rossier BC (2014). Epithelial sodium channel (ENaC) and the control of blood pressure. Curr Opin Pharmacol.

[51] Rossier BC, Stutts MJ (2009). Activation of the epithelial sodium channel (ENaC) by serine proteases. Annu Rev Physiol.

[52] Ruffieux-Daidie D, Staub O (2011). Intracellular ubiquitylation of the epithelial Na^+^ channel controls extracellular proteolytic channel activation via conformational change. J Biol Chem.

[53] Ruffieux-Daidie D, Poirot O, Boulkroun S, Verrey F, Kellenberger S, Staub O (2008). Deubiquitylation regulates activation and proteolytic cleavage of ENaC. J Am Soc Nephrol.

[54] Saxena S, Quick MW, Tousson A, Oh Y, Warnock DG (1999). Interaction of syntaxins with the amiloride-sensitive epithelial sodium channel. J Biol Chem.

[55] Saxena SK, George CM, Pinskiy V, McConnell B (2006). Epithelial sodium channel is regulated by SNAP-23/syntaxin 1A interplay. Biochem Biophys Res Commun.

[56] Saxena SK, Singh M, Kaur S, George C (2007). Distinct domain-dependent effect of syntaxin1A on amiloride-sensitive sodium channel (ENaC) currents in HT-29 colonic epithelial cells. Int J Biol Sci.

[57] Seebohm G, Strutz-Seebohm N, Birkin R, Dell G, Bucci C, Spinosa MR, Baltaev R, Mack AF, Korniychuk G, Choudhury A, Marks D, Pagano RE, Attali B, Pfeufer A, Kass RS, Sanguinetti MC, Tavare JM, Lang F (2007). Regulation of endocytic recycling of KCNQ1/KCNE1 potassium channels. Circ Res.

[58] Seebohm G, Neumann S, Theiss C, Novkovic T, Hill EV, Tavare JM, Lang F, Hollmann M, Manahan-Vaughan D, Strutz-Seebohm N (2012). Identification of a novel signaling pathway and its relevance for GluA1 recycling. PLoS One.

[59] Shimkets RA, Lifton RP, Canessa CM (1997). The activity of the epithelial sodium channel is regulated by clathrin-mediated endocytosis. J Biol Chem.

[60] Snyder PM, Olson DR, Thomas BC (2002). Serum and glucocorticoid-regulated kinase modulates Nedd4-2-mediated inhibition of the epithelial Na^+^ channel. J Biol Chem.

[61] Staub O, Gautschi I, Ishikawa T, Breitschopf K, Ciechanover A, Schild L, Rotin D (1997). Regulation of stability and function of the epithelial Na^+^ channel (ENaC) by ubiquitination. EMBO J.

[62] Stewart AP, Haerteis S, Diakov A, Korbmacher C, Edwardson JM (2011). Atomic force microscopy reveals the architecture of the epithelial sodium channel (ENaC). J Biol Chem.

[63] Strutz-Seebohm N, Korniychuk G, Schwarz R, Baltaev R, Ureche ON, Mack AF, Ma ZL, Hollmann M, Lang F, Seebohm G (2006). Functional significance of the kainate receptor GluR6(M836I) mutation that is linked to autism. Cell Physiol Biochem.

[64] Teng FY, Wang Y, Tang BL (2001) The syntaxins. Genome Biol 2:REVIEWS3012. 10.1186/gb-2001-2-11-reviews3012 10.1186/gb-2001-2-11-reviews3012 PMC138984 11737951

[65] Volk T, Konstas AA, Bassalay P, Ehmke H, Korbmacher C (2004). Extracellular Na^+^ removal attenuates rundown of the epithelial Na^+^-channel (ENaC) by reducing the rate of channel retrieval. Pflugers Arch.

[66] Ware AW, Rasulov SR, Cheung TT, Lott JS, McDonald FJ (2020). Membrane trafficking pathways regulating the epithelial Na^+^ channel. Am J Physiol Renal Physiol.

[67] Warnock DG (1998). Liddle syndrome: an autosomal dominant form of human hypertension. Kidney Int.

[68] Zerangue N, Schwappach B, Jan YN, Jan LY (1999). A new ER trafficking signal regulates the subunit stoichiometry of plasma membrane K_ATP_ channels. Neuron.

[69] Zhou R, Patel SV, Snyder PM (2007). Nedd4-2 catalyzes ubiquitination and degradation of cell surface ENaC. J Biol Chem.

